# Hericioic Acids **A–G** and Hericiofuranoic Acid; Neurotrophic Agents from Cultures of the European Mushroom *Hericium flagellum*

**DOI:** 10.1021/acs.jafc.3c02897

**Published:** 2023-07-13

**Authors:** Winnie Chemutai Sum, Sherif S. Ebada, Marco Kirchenwitz, Harald Kellner, Mahmoud A. A. Ibrahim, Theresia E. B. Stradal, Josphat Clement Matasyoh, Marc Stadler

**Affiliations:** † Department of Microbial Drugs, Helmholtz Centre for Infection Research GmbH (HZI), Inhoffenstraße 7, 38124 Braunschweig, Germany; ‡ Institute of Microbiology, Technische Universität Braunschweig, Spielmannstraße 7, 38106 Braunschweig, Germany; § Department of Pharmacognosy, Faculty of Pharmacy, Ain Shams University, 11566 Cairo, Egypt; ∥ Department of Cell Biology, Helmholtz Centre for Infection Research, Inhoffenstrasse 7, 38124 Braunschweig, Germany; ⊥ Department of Bio- and Environmental Sciences, Technische Universität Dresden-International Institute Zittau, Markt 23, 02763 Zittau, Germany; # Computational Chemistry Laboratory, Chemistry Department, Faculty of Science, Minia University, 61519 Minia, Egypt; ¶ School of Health Sciences, University of KwaZulu-Natal, Westville, 4000 Durban, South Africa; ∇ Department of Chemistry, Egerton University, P.O. Box 536, 20115 Njoro, Kenya

**Keywords:** Basidiomycota, Hericiaceae, isoindolinone, benzofuranone, neurotrophic

## Abstract

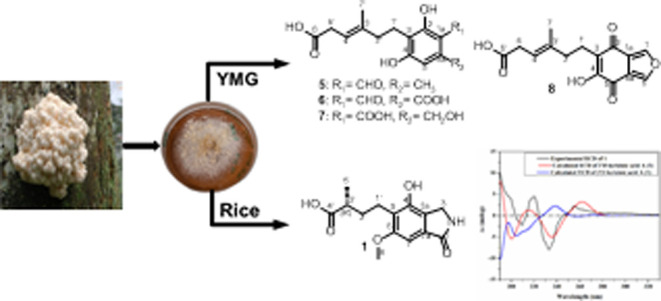

Neurodegenerative diseases are currently posing huge social, economic, and healthcare burdens among the aged populations worldwide with few and only palliative treatment alternatives available. Natural products continue to be a source of a vast array of potent neurotrophic molecules that could be considered as drug design starting points. The present study reports eight new isoindolinone and benzofuranone derivatives, for which we propose the trivial names, hericioic acids **A–G** (**1**–**7**) and hericiofuranoic acid (**8**), which were isolated from a solid culture (using rice as substrate) of the rare European edible mushroom *Hericium flagellum*. The chemical structures of these compounds were determined based on extensive 1D and 2D NMR spectroscopy along with HRESIMS analyses. The isolated compounds were assessed for their neurotrophic activity in rat pheochromocytoma cells (PC-12) to promote neurite outgrowth on 5 ng NGF supplementation; all the compounds increased neurite outgrowths, with compounds **3**, **4,** and **8** exhibiting the strongest effects.

## 1 Introduction

Neurotrophic factors such as brain-derived neurotrophic factor (BDNF), nerve growth factor (NGF), glial-derived neurotrophic factor (GDNF), and neurotrophin-3 are secreted by the glial cells to support regeneration, functionality, maintenance, and survival of neuronal cells.^1,2^ In addition, the interaction between other cell types and neurotrophins enhances various physiological activities, hence extending their roles far beyond the horizon of the central and peripheral nervous system.^3^ The decreased levels of neurotrophins have been linked to neurodegenerative diseases (NDDs), such as Huntington’s, Alzheimer’s, and Parkinson’s diseases that affects about 50 million of the aged population worldwide.^4,5^ Currently, the healthcare-related impacts of NDDs remain unmet with very few interventions to induce nerve regeneration. The major bottlenecks are the inability of neurotrophic factors to cross the blood–brain barrier due to high molecular weight and their easy degradation by peptidases.^5^ Thus, the discovery of alternative low-molecular weight NGF-substitutes and/or NGF-inducing molecules comes in handy to combat NDDs.

For centuries, basidiomycetes of the genus *Hericium* (Hericiaceae) have been used as medical provisions and/or highly valuable food^6^ and their metabolites were shown to exhibit various biological activities.^7^ Notably, the isolated meroterpenoids and terpenoids like hericenones, corallocins, and erinacines have been shown to stimulate the production of NGF and/or BDNF in cell-based assays.^6−8^ The most widely studied edible mushroom in the fungal genus is the famous Lion’s Mane, *Hericium erinaceus,* which is widely employed in Chinese traditional medicine as well as food and grown commercially in ton scale.^6^ Interestingly, the fungus has also recently drawn attention due to the probable application of its secondary metabolites in the development of therapeutic and neuroprotective interventions.^7−9^

In our continued search for neuroprotective agents, we investigated the species *Hericium flagellum* (also known under the younger synonym *Hericium alpestre*); a highly host-dependent rare, red-listed species majorly found on silver fir (*Abies alba*).^10^ It mainly occurs in undisturbed forests and is not as widely studied as its relatives.^11^ So far, the only compounds isolated from *H. flagellum* are cyathane diterpenoids, which were obtained from liquid cultures using a strain that had been deposited many years in a public collection.^8^ In the current study, we have examined a newly isolated strain and also checked the metabolite production on solid media. We have detected several previously unknown peaks by HPLC-MS analytics in the rice cultures of the fungus and decided to isolate the corresponding compounds. The current paper deals with their structure elucidation and biological characterization.

## 2 Materials and Methods

### 2.1 General Experimental Procedures

The compounds were dissolved in deuterated solvents (CD_3_OD and (CD_3_)_2_SO), and their 1D and 2D NMR spectra recorded on Avance III 700 (Daltonics, Bremen, Germany; ^1^H 700 MHz, ^13^C 175 MHz) or Avance III 600 (Bruker, ^1^H 600 MHz, ^13^C 150 MHz) spectrometers. Coupling constants were calculated in Hertz (Hz), whereas chemical shifts were obtained in parts per million (ppm). UV/vis spectra were recorded on a Shimadzu UV/vis 2450 spectrophotometer (Kyoto, Japan), at a concentration of 0.02 mg/mL using methanol or dimethyl sulfoxide as solvents. IR spectra were recorded on a PerkinElmer FT-IR, Spectrum 100 spectrometer. ECD spectra were obtained with a Jasco J-815 spectropolarimeter at a concentration of 0.02 mg/mL using dimethyl sulfoxide as solvent (JASCO, Pfungstadt, Germany).

HPLC-DAD/MS of the yeast and malt extract with glucose (YMG) and rice media crude extracts of *H. flagellum* were analyzed on the amaZon speed ETD ion trap mass spectrometer (Bruker) in the positive and negative ionization modes. The HPLC system consisted of a Dionex UltiMate 3000 UHPLC (Thermo Fisher Scientific Inc, Waltham, MA, USA) equipped with a C18 Acquity UPLC BEH column (Waters, Milford, USA). The eluents were solvent A: deionized water + 0.1% formic acid (FA) (v/v) and solvent B: acetonitrile + 0.1% FA (v/v). The gradient system was as follows: 5% B for 0.5 min, increasing to 100% B over a period of 20 min and maintaining at 100% B for 10 min, at a flow rate of 0.6 mL/min and UV/vis detections at 190–600 nm and 210 nm wavelengths. HR-(+) ESIMS data were analyzed on a maXis ESI-TOF (Time of Flight) mass spectrometer (Bruker) connected to an Agilent 1260 series HPLC-UV system (Agilent Technologies, Santa Clara, CA, USA) equipped with a C18 Acquity UPLC BEH column (Waters). The eluents were solvent A: deionized water + 0.1% formic acid (FA) (v/v) and solvent B: acetonitrile + 0.1% FA (v/v). The gradient system was as follows: 5% B for 0.5 min, increasing to 100% B over a period of 19.5 min, and maintaining at 100% B for 5 min, at a flow rate of 0.6 mL/min at 40 °C and UV/Vis detection at 200–600 nm. The molecular formulas of the compounds detected were then calculated using the Smart Formula algorithm of the Compass Data Analysis software (Bruker, version 4.4).

The chemicals and solvents (analytical and HPLC grade) used were sourced from AppliChem GmbH (Darmstadt, Germany), Carl Roth GmbH & Co. KG (Karlsruhe, Germany), Avantor Performance Materials (Deventer, Netherlands), and Merck (Darmstadt, Germany). Preparation of deionized water was done on the PURELAB flex water purification system (Veolia Water Technologies, Celle, Germany).

### 2.2 Fungal Material and Fermentation

The fruiting body of *H. flagellum* was collected from an *A. alba* log in a coniferous forest at the Zwieseler Waldhaus in the Bavarian Forest National Park (49.098387 N, 13.246003 E), Germany, in August 2015 by one of the authors (H.K.). A voucher specimen and mycelial cultures are stored in German culture collection (DSMZ), Braunschweig, under the accession number DSM108284. A genome sequence of this strain was also generated and is deposited with GenBank at the following URL: https://www.ncbi.nlm.nih.gov/assembly/GCA_004681135.1#/st.

Preparation of the *H. flagellum* seed cultures was done following previously laid out procedure^8^ with slight modification. Five mycelial plugs (5 mm diameter) were cut out from fully grown YMG agar plates (prepared by dissolving yeast extract 4 g, glucose 4 g, malt extract 10 g, and agar 20 g in 1L deionized water). These were inoculated into two culture flasks containing 100 mL YMG liquid media. The flasks were incubated at 24 °C at 160 rpm shaking conditions for 14 days. These were then homogenized and 10mL mycelial suspension used to inoculate twenty 500 mL Erlenmeyer flasks each containing 200 mL YMG. The inocula were incubated in the dark at 24 °C and 160 rpm on a rotary shaker for 90 days. The rice medium cultures were prepared according to Chepkirui et al.^12^ Basically, 90 g rice in 90 mL distilled water were prepared in ten 500 mL Erlenmeyer flasks and autoclaved. The inoculation of fungal mycelial plugs into the solid medium was done as previously performed for the liquid YMG medium. The cultures were then incubated under static conditions at 24 °C for 90 days.

### 2.3 Extraction and Isolation of Compounds

The liquid-state fermentation cultures were extracted as previously elaborated.^13^ Basically, the cultures were filtered and the mycelia extracted with acetone. The supernatant portion was extracted with 1% (m/v) of Amberlite XAD 16N adsorber resin (Sigma-Aldrich, Deisenhofen, Germany). The resin was then extracted with acetone in a similar manner to the mycelia, and the solvent evaporated from the filtrate. The remaining semi-dried portion was reconstituted in distilled water and partitioned with an equal volume of ethyl acetate. The organic phase was filtered through anhydrous sodium sulfate and dried in a rotary evaporator (Heidolph, Schwabach) to yield 2.0 and 0.6 g of supernatant and mycelial crude extracts, respectively. Similarly, the solid-state fermentation cultures were extracted twice with acetone. Initially, they were soaked overnight in acetone with mild shaking (120 rpm). The solvent was then evaporated, and the filtrate was partitioned with ethyl acetate and dried likewise to the YMG cultures to afford 6.4 g of crude extract.

The HPLC-DAD/MS analyses of ethyl acetate crude extracts obtained from *H. flagellum* rice and YMG culture media were performed and this showed the presence of hitherto undescribed compounds. Therefore, ethyl acetate extracts were purified and their included secondary metabolites were isolated using a preparative HPLC (PLC 2020; Gilson, Middleton, WI, USA) system. The eluents used were solvent A: deionized water + 0.1% FA (v/v) and solvent B: ACN + 0.1% FA (v/v). C_18_ VP-Nucleodur column 100–5 (250 mm × 40 mm, 7 μm: Machery-Nagel, Düren, Germany) was used as the stationary phase. The flow rate was 40 mL/min, and the UV detections were obtained at 190, 210 and 280 nm wavelengths.

The separation gradient used to isolate compounds obtained from the liquid-state fermentation was as follows: initial 5% B for 10 min, followed by 5–15% B within 3 min, thereafter 15–60% B for 50 min, an increase from 60 to 100%B within 5 min and finally holding the gradient at 100% B for 5 min to yield **7** (1.7 mg, *t*_R_ = 30 min), **6** (8.5 mg, *t*_R_ = 40 min), **8** (2.9 mg, *t*_R_ = 43 min), and **5** (1.4 mg, *t*_R_ = 53 min).

Similarly, the solid-state fermentation compounds were eluted by the following: initial holding at 5% for 10 min, 5–20% B for 5 min, 20–50% B within 50 min, 50–100% B within 5 min, and holding for 5 min at 100% B, to yield compounds **1** (6.9 mg, *t*_R_ = 43 min), **2** (6.8 mg, *t*_R_ = 41 min), **3** (11.2 mg, *t*_R_ = 36 min), and **4** (23.3 mg, *t*_R_ = 52 min).

### 2.4 Cytotoxicity Tests

In vitro cytotoxic effects of the compounds were evaluated at an initial concentration of 1 mg/mL followed by a serial dilution based on an established protocol as previously reported.^14^

### 2.5 Preparation of Cell Cultures

The adherent variant rat pheochromocytoma cells (PC-12) were sourced from the European Collection of Authenticated Cell Cultures (ECACC). These were cultured in Gibco RPMI-1640 (Fisher Scientific, Hampton, NH, USA) media consisting of 5% heat-inactivated fetal bovine serum (FBS) (Capricorn) and 10% horse serum (Capricorn Scientific GmbH, Ebsdorfergrund, Germany). Likewise, the astrocytoma (1321N1) cells were obtained from Sigma-Aldrich (acc. no. 86030402) and cultured in Gibco DMEM media (Fisher Scientific, Inc., Waltham, MA, USA) containing 10% heat-inactivated FBS (Capricorn).^15^ The media for culturing 1321N1 cells was supplemented with streptomycin (86 μM), glutamine (2 mM), and penicillin (0.15 mM).^15^ The incubation of the cells was performed in a humidified environment of 7.5% CO_2_ at 37 °C. The cells were passaged 3–4 days. Collagen type IV (Sigma C5533) was coated on 96-well plates and left for 6 h before using the plates when seeding PC-12 cells.

### 2.6 Neurite Outgrowth Assays

Neurotrophic activity tests were conducted, as previously established.^16^ Basically, PC-12 cells were seeded on collagen type IV (C5533, Sigma-Aldrich, St. Louis, MO, USA)-coated 96-well plates at a density of 1.5 × 10^4^ cells per well and incubated for 6 h to cells attached to the culture vessel. The cells were treated with compounds in differentiation media (RPMI-1640, 1% heat-inactivated horse serum, 2 mM l-glutamine and 1 × P/S) in the presence and absence of NGF (5 ng/mL). The treatment with DMSO served as a vehicle control. Neurite outgrowth was examined by phase contrast imaging after 48 h via an IncuCyte S3 live-cell analysis system (Sartorius, Göttingen, Germany). IncuCyte NeuroTrack Software Module was used to measure the neurite lengths.

### 2.7 Statistical Analysis

The data were analyzed on Prism V8 software (GraphPad Software Inc., San Diego, CA, USA) employing the student *t*-test statistical method. Data are displayed as the mean ± SEM.

### 2.8 Computational Functional Theory Calculations

A conformational analysis was principally executed to reveal all plausible conformations of hericioic acid **A** (**1**) within an energy window value of 10 kcal/mol using Omega2 software.^17^ The resulting conformers were geometrically optimized and then subjected to frequency computation at the B3LYP/6-31G* level of theory with the help of Gaussian09 software.^18^ Upon the obtained data from frequency calculations, the Gibbs free energies were calculated. With the incorporation of the polarizable continuum model, the time-dependent density functional theory calculations were carried out employing dimethylsulfoxide as a solvent to determine the excitation states (*n* = 50). Electronic circular dichroism (ECD) spectra were graphed using the SpecDis 1.71 software with a sigma value of 0.20–30 eV.^19,20^ The extracted ECD spectra were Boltzmann-averaged.

## 3 Results and Discussion

**Figure 1:**
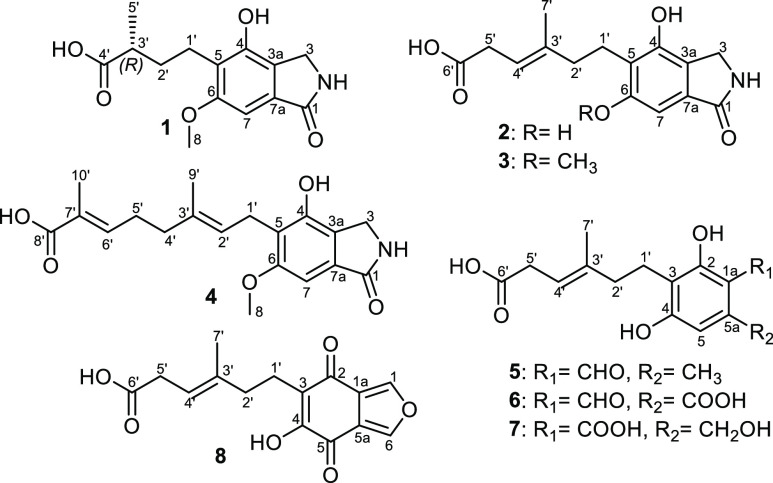
Chemical structures of **1**–**8**.

HPLC-DAD/MS analyses of the YMG and rice media ethyl acetate extracts revealed the presence of new secondary metabolites, which were isolated and purified as detailed in Section 2.3. Eight new isoindolinone and benzofuranone structures were successfully elucidated based on the interpretation of extensive NMR measurements coupled with HR-ESIMS data (Figure 1).

Compound **1** was isolated as an off-white amorphous solid and its molecular formula was established to be C_14_H_17_NO_5_ based on HRESIMS that revealed a pseudomolecular ion peak at *m/z* 280.1176 [M + H]^+^ (calculated 280.1179) indicating its inclusion to seven degrees of unsaturation in its structure. The ^13^C NMR spectrum of **1** (Table 1) exhibited the existence of 14 different carbon resonances distinguished into seven quaternary carbons including two carbonyl (δ_C_ 177.5 (C-4′), 170.3 (C-1)), two oxygenated olefinic (δ_C_ 158.5 (C-6), 150.4 (C-4)), and three olefinic carbons (δ_C_ 131.7 (C-7a), 122.7 (C-3a), 120.2 (C-5)) along with two tertiary carbons (δ_C_ 96.1 (C-7), 38.7 (C-3′)), three secondary carbons (δ_C_ 43.0 (C-3), 32.6 (C-2′), 20.8 (C-1′)), one methoxy (δ_C_ 55.8 (C-8)), and one aliphatic methyl carbon (δ_C_ 16.9 (C-5′)).

**Table 1:** ^1^H and ^13^C NMR Data of **1**–**4**

	**1**		**2**		**3**		**4**	
pos.	δ_C_,^a^^,^^c^ type	δ_H_^b^ (multi, *J* [Hz])	δ_C_,^a^^,^^c^ type	δ_H_^b^ (multi, *J* [Hz])	δ_C_,^a^^,^^c^ type	δ_H_^b^ (multi, *J* [Hz])	δ_C_,^a^^,^^c^ type	δ_H_^b^ (multi, *J* [Hz])
1	170.3, CO		170.5, CO		170.4, CO		170.4, CO	
3	43.0, CH_2_	4.18 (br s, 2H)	42.9, CH_2_	4.13 (br s, 2H)	43.0, CH_2_	4.19 (br s, 2H)	43.1, CH_2_	4.19 (br s, 2H)
3a	123.1, C		121.0, C		123.1, C		123.2, C	
4	150.4, C		150.5, C		150.3, C		150.2, C	
5	120.2, C		119.2, C		120.3, C		119.7, C	
6	158.5, C		156.3, C		158.5, C		158.4, C	
7	96.1, CH	6.73 (s, 1H)	100.2, CH	6.63 (s, 1H)	96.2, CH	6.73 (s, 1H)	96.3, CH	6.73 (s, 1H)
7a	131.7, C		131.3, C		131.6, C		131.6, C	
8	55.8, CH_3_	3.79 (s, 3H)			55.8, CH_3_	3.80 (s, 3H)	55.8, CH_3_	3.79 (s, 3H)
1′	20.8, CH_2_	2.64 (t, 7.7, 2H)	22.1, CH_2_	2.68 (t, 8.0, 2H)	21.9, CH_2_	2.72 (t, 8.0, 2H)	22.4, CH_2_	3.32 (d, 7.2, 2H)
2′	32.6, CH_2_	*α* 1.44 (dq, 13.2, 7.5, 1H)	38.2, CH_2_	2.09 (t, 8.0, 2H)	38.3, CH_2_	2.08 (t, 8.0, 2H)	122.8, CH	5.15 (td, 7.2, 1.3, 1H)
		β 1.74 (dq, 13.2, 7.4, 1H)						
3′	38.7, CH	2.27 (h, 7.0, 1H)	138.1, C		137.8, C		133.6, C	
4′	177.5, CO		116.2, CH	5.29 (td, 7.0, 1.4, 1H)	116.4, CH	5.25 (td, 7.0, 1.4, 1H)	37.8, CH_2_	2.01 (t, 7.5, 2H)
5′	16.9, CH_3_	1.10 (d, 7.0, 3H)	33.3, CH_2_	2.96 (d, 7.0, 2H)	33.3, CH_2_	2.95 (d, 6.0, 2H)	26.8, CH_2_	2.20 (q, 7.5, 2H)
6′			173.2, CO		173.2, CO		141.3, CH	6.58 (td, 7.5, 1.5, 1H)
7′			16.4, CH_3_	1.66 (d, 1.4, 3H)	16.4, CH_3_	1.65 (d, 1.4, 3H)	127.7, C	
8′							168.9, CO	
9′							15.9, CH_3_	1.74 (d, 1.3, 3H)
10′							12.3, CH_3_	1.69 (d, 1.5, 3H)
2-N*H*		8.35 (br s, 1H)		8.28 (br s, 1H)		8.37 (br s, 1H)		8.37 (br s, 1H)
4-O*H*		9.46 (br, 1H)		9.17 (br s, 1H)		9.42 (br, 1H)		9.42 (br s, 1H)
6-O*H*				9.47 (br s, 1H)				
4′/6′/8′-COOH		12.05 (br, 1H)		12.11 (br s, 1H)		12.05 (br, 1H)		12.10 (br, 1H)

a Measured in DMSO-*d*_6_ at 150 MHz.

b Measured in DMSO-*d*_6_ at 600 MHz.

c Assigned based on HMBC and HSQC spectra.

**Figure 2:**
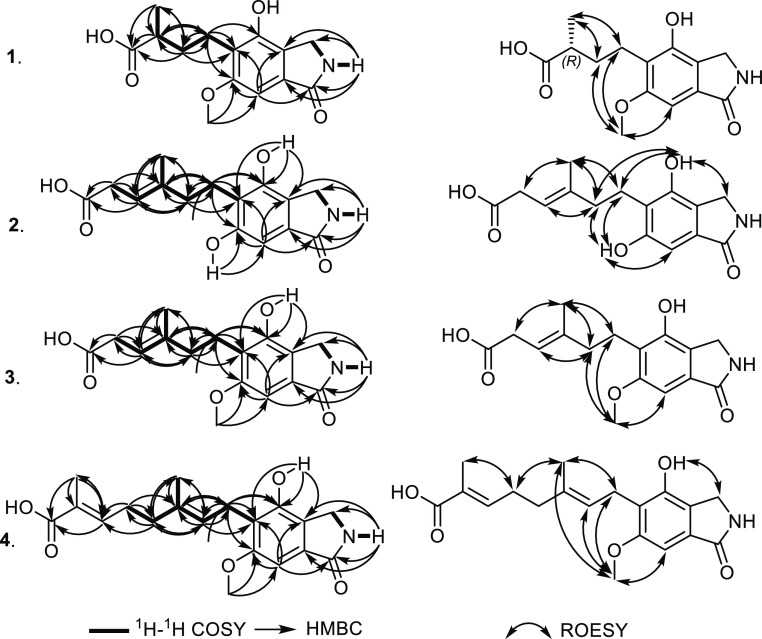
Key ^1^H–^1^H COSY, HMBC, and ROESY correlations of **1**–**4**.

**Figure 3:**
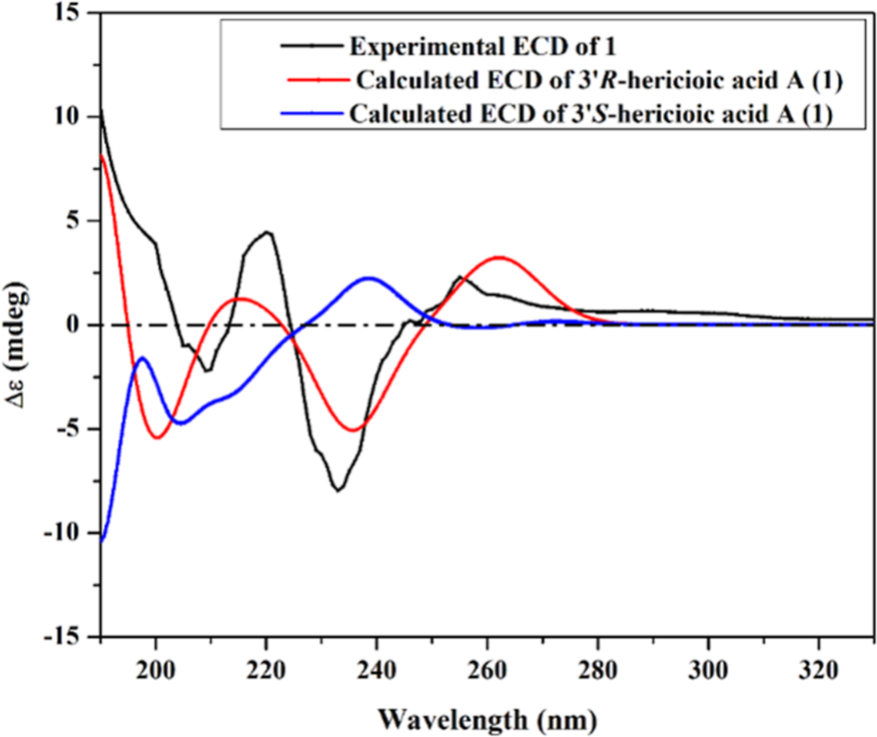
Experimental and calculated **ECD** spectrum of **1**.

The ^1^H NMR and HSQC of **1** (Table 1) displayed the presence of a deshielded exchangeable proton at δ_H_ 8.35 (H-2) that was found to be correlated in ^1^H–^1^H COSY spectrum (Figure 2) to a methylene group at δ_H_ 4.18 (H_2_-3). The ^1^H–^1^H COSY spectrum of **1** (Table 1, Figure 2) showed the presence of a second spin system extending over two methylene groups at δ_H_ 2.64 (t, *J* = 7.7 Hz, H_2_-1′), δ_H_ 1.74 (dq, *J* = 7.4, 13.2 Hz, Ha-2′), δ_H_ 1.44 (dq, *J* = 7.5, 13.2 Hz, Hb-2′) and further to a multiplet proton at δ_H_ 2.27 (m, *J* = 7.0 Hz, H_2_-3′), ending by a doublet methyl resonance at δ_H_ 1.10 (d, *J* = 6.9 Hz, H_3_-5′). Based on the obtained results and by comparison with the reported metabolites from the genus *Hericium*, compound **1** was found to resemble the isoindolinone fragment of *N*-De phenylethyl isohericerin.^6,21^ The HMBC spectrum of **1** (Figure 2) showed key correlations from both protons at δ_H_ 8.35 (N*H*-2) and at δ_H_ 4.18 (H_2_-3) to one carbonyl carbon (δ_C_ 170.3, C-1), two quaternary carbons at δ_C_ 122.7 (C-3a) and δ_C_ 131.7 (C-7a), whereas the latter also revealed a key correlations to an oxygenated aromatic carbon (δ_C_ 150.4, C-4) confirming the existence of isoindolinone moiety. The position of the methoxy group at δ_H_ 3.79 (s, H_3_-8; δ_C_ 55.8) was confirmed by HMBC spectrum that revealed its key correlations together with an aromatic singlet proton at δ_H_ 6.73 (s, H-7) to the same oxygenated aromatic carbon (δ_C_ 158.5, C-6). The major structural difference between **1** and *N*-De phenylethyl isohericerin was notified in their side chains. The ^1^H–^1^H COSY and ROESY spectra of **1** (Figure 2) confirmed its side chain to be −(CH_2_)_2_–CHCH_3_–COOH, whereas HMBC spectrum proved it to be located at C-5 (δ_C_ 120.2) supported by its key correlations with two methylene groups at δ_H_ 2.64 (H_2_-1′) and at δ_H_ 1.74/1.44 (H_2_-2′). The presence of a terminal carboxylic acid group at the end of the side chain was confirmed by key HMBC correlations from H_2_-1′, H_2_-2′ and a doublet methyl group (H_3_-5′) to a carboxylic acid group (δ_C_ 177.5, C-4′). The ROESY spectrum of **1** (Figure 2) also exhibited key NOE correlations from methoxy group (H_3_-8) to H-7, H_2_-1′and H_2_-2′. To determine the absolute configuration of C-3′, the ECD spectrum of **1** (Figure 3) was measured and compared to the calculated ECD spectra of both *R* and *S* isomers. The measured and calculated ECD spectra of 3′*R*-isomer revealed a close accordance over the whole range that unambiguously assigns C-3′ to be in *R*-configuration. Based on the aforementioned results, the chemical structure of **1** was undoubtedly determined to be an isoindolinone derivative as depicted in Figure 1 and it was given a trivial name hericioic acid A.

Compound **2** was obtained as a white amorphous solid and its molecular formula was determined to be C_15_H_17_NO_5_ by HRESIMS that revealed a pseudomolecular ion peak at *m/z* 292.1179 [M + H]^+^ (calculated 292.1179), indicating the existence of eight degrees of unsaturation. The ^1^H NMR and ^13^C NMR spectral data of **2** (Table 1) revealed a similar presence of isoindolinone moiety as in **1** with two main differences, namely, the absence of a methoxy group and the presence of an extra methylene group on the aliphatic side chain in **2** compared to **1**. The ROESY spectrum of **2** (Figure 2) revealed key NOE correlations from two exchangeable protons at δ_H_ 9.17 (br s, 4-O*H*) and δ_H_ 9.47 (br s, 6-O*H*) to a methylene group at δ_H_ 4.13 (br d, *J* = 1.6 Hz, H_2_-3) and an aromatic proton at δ_H_ 6.63 (H-7), respectively, indicating the two hydroxyl groups to be positioned at C-4 and C-6, respectively. The ^1^H–^1^H COSY spectrum of **2** (Figure 2) revealed an extended spin system over two methylene groups at δ_H_ 2.68 (H_2_-1′) and at δ_H_ 2.09 (dd, *J* = 6.2, 10.1 Hz, H_2_-2′) to an olefinic proton at δ_H_ 5.29 (tq, *J* = 1.4, 7.1 Hz, H-4′) ending at an additional downfield methylene group at δ_H_ 2.96 (d, *J* = 6.9, 10.1 Hz, H_2_-5′). It also revealed the presence of an olefinic methyl group at δ_H_ 1.66 (d, *J* = 1.3 Hz, H_3_-7′). In addition, HSQC and HMBC spectra of **2** (Figure 2) unambiguously confirmed that the aliphatic side chain is similarly located at C-5 as in **1** based on the key correlations from two methylene groups at δ_H_ 2.68 (m, H_2_-1′) and at δ_H_ 2.09 (dd, *J* = 6.2, 10.1 Hz, H_2_-2′) to a quaternary aromatic carbon at δ_C_ 119.2 (C-5). The HMBC spectrum also exhibited key correlations from H_3_-7′ to two olefinic carbons at δ_c_ 138.1 (C-3′) and δ_C_ 116.2 (C-4′) and similar to **1** confirmed the presence of a terminal carboxylic acid group by the key HMBC correlations from H-4′ and H_2_-5′ to a carboxylic acid carbon at δ_C_ 173.2 (C-6′). Therefore, compound **2** was confirmed to be a new isoindolinone derivative featuring a terminal carboxylic acid moiety that was named as hericioic acid B.

Compound **3** was purified as a white amorphous solid whose molecular formula was established to be C_16_H_19_NO_5_ based on HRESIMS showing a pseudomolecular ion peak at *m/z* 306.1334 [M + H]^+^ (calculated 306.1336), indicating equal degrees of unsaturation as in **2** with a molecular difference of 14 amu assigned probably to an additional CH_2_ in its molecular formula. This difference was obviously interpreted by comparing ^1^H and ^13^C NMR data of **2** and **3** (Table 1) that clearly showed the presence of a methoxy group in **3** at δ_H_ 3.80 (H_3_-8; δ_C_ 55.8) instead of a hydroxyl group in **2**. The HMBC spectrum of **3** unambiguously displayed clear correlations from the methoxy group and an aromatic proton at δ_H_ 6.73 (H-7) to an oxygenated aromatic carbon at δ_C_ 158.5 (C-6). Therefore, the chemical structure of **3** was identified as 6-*O*-methyl derivative of **2** and was named as hericioic acid C.

Compound **4** was obtained as a yellow amorphous solid where its HRESIMS revealed a pseudomolecular ion peak at *m/z* 346.1649 [M + H]^+^ establishing its molecular formula to be C_19_H_23_NO_5_ (calculated 346.1649) indicating the existence of nine degrees of unsaturation. By comparing ^1^H and ^13^C NMR data of **4** (Table 1) to those reported for *N*-De phenylethyl isohericerin,^21^ an obvious resemblance can be noticed with only one difference in the presence of one carbonyl carbon at δ_C_ 168.9 instead of an olefinic methyl group. The ^1^H–^1^H COSY spectrum of **4** revealed an extended spin system over a methylene group at δ_H_ 3.32 (d, *J* = 7.2 Hz, H_2_-1′), an olefinic proton at δ_H_ 5.15 (tq, *J* = 1.3, 7.2 Hz, H-2′), two methylene groups at δ_H_ 2.01 (t, *J* = 7.5 Hz, H_2_-4′), δ_H_ 2.20 (q, *J* = 7.5 Hz, H_2_-5′), and an olefinic proton at δ_H_ 6.58 (tq, *J* = 1.5, 7.3 Hz, H-6′). The ^1^H–^1^H COSY spectrum of **4** also revealed the presence of two olefinic methyl groups at δ_H_ 1.74 (d, *J* = 1.3 Hz, H_3_-9′) and δ_H_ 1.69 (d, *J* = 1.4 Hz, H_3_-10′) that were spin-coupled to two olefinic protons H-2′ and H-6′, respectively. The HSQC spectrum of **4** revealed a clear direct correlation of H-6′ to a highly downfield carbon (δ_C_ 141.3, C-6′) and the olefinic methyl group H_3_-10′ to be directly correlated with an upfield carbon (δ_C_ 12.3, C-10′). In addition, HMBC spectrum of **4** (Figure 2) showed clear correlations from H_3_-10′ to carbon resonances C-6′ (δ_C_ 141.3), C-7′ (δ_C_ 127.7), and C-8′ (δ_C_ 168.9) suggesting its existence as an α-substituent on an α,β-unsaturated carboxylic acid moiety. The ROESY spectrum of **4** disclosed similar key NOE correlations to hericioic acids **A** (**1**) and **C** (**3**) such as those from a methoxy group H_3_-8 to H-7, H_2_-1′, and H_2_-2′ along with other key NOE correlations from two olefinic methyl groups H_3_-9′ and H_3_-10′ to methylene group H_2_-5′ confirming the depicted structure of **4** and it was trivially named as hericioic acid D.

**Figure 4:**
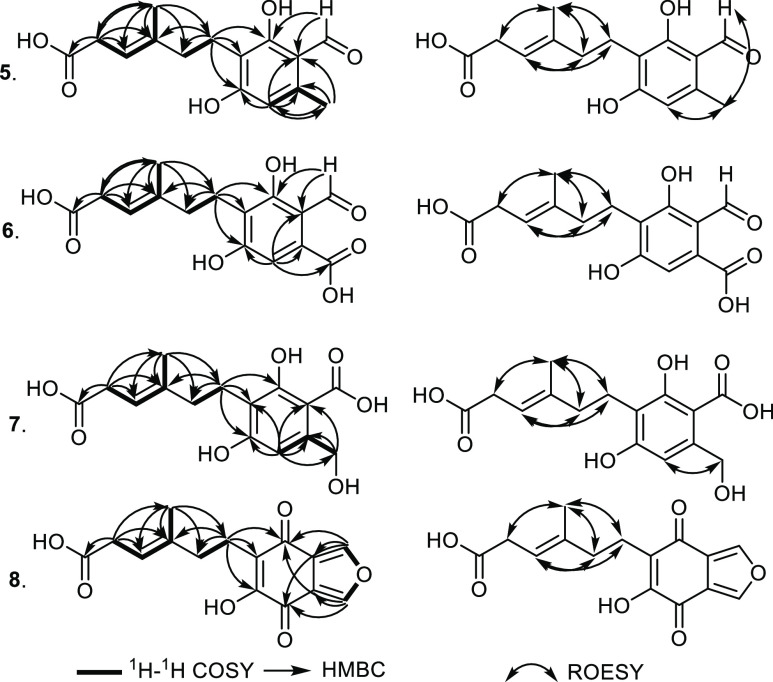
Key ^1^H–^1^H COSY, HMBC, and ROESY correlations of **5–8**.

Compound **5** was purified as a brown oil and its molecular formula was determined to be C_15_H_18_O_5_ based on its HRESIMS that showed pseudomolecular ion peaks at *m/z* 279.1228 [M + H]^+^ (calculated for 279.1227) and at *m/z* 301.1048 [M + Na]^+^ (calculated for 301.1046) indicating the presence of seven degrees of unsaturation. The ^1^H and ^13^C NMR spectral data of **5** (Table 2) revealed comparable values to those reported in literature for 4-(3′,7′-dimethyl-2′,6′-octadienyl)-2-formyl-3-hydroxy-5-methoxybenzylalcohol, alcoholic moiety incorporated in hericenes **A–C**(22) and as a natural metabolite of *H. erinaceus* (as “erinaceum”).^23^ Careful comparison of ^1^H and ^13^C NMR spectral values of **5** with those reported in literature^22,23^ disclosed some major differences in particular the absence of a methoxy and a hydroxymethylene moieties, the presence of an aromatic methyl group at δ_H_ 2.47 (d, *J* = 0.7 Hz, H_3_-7) and its aliphatic side chain features different lengths and functional groups compared to those reported in literature.^22,23^ The ^1^H–^1^H COSY and HSQC spectra of **5** (Figure 4) showed three different spin systems, namely, between an aromatic methyl group at δ_H_ 2.47 (d, *J* = 0.7 Hz, H_3_-7; δ_C_ 18.1) to an aromatic proton peak at δ_H_ 6.22 (d, *J* = 0.9 Hz, H-5; δ_C_ 111.0); between two aliphatic methylene groups at δ_H_ 2.69 (H_2_-1′; δ_C_ 21.8) and at δ_H_ 2.19 (H_2_-2′; δ_C_ 39.5); between an olefinic proton at δ_H_ 5.28 (ddt, *J* = 1.4, 4.1, 7.2 Hz, H-4′; δ_C_ 117.5) showing vicinal and allylic coupling to a methylene group at δ_H_ 2.98 (dd, *J* = 1.2, 7.3 Hz, H_2_-5′; δ_C_ 34.6) and a methyl peak at δ_H_ 1.73 (d, *J* = 1.4 Hz, H_3_-7′; δ_C_ 16.3), respectively. In addition, the HSQC spectrum of **5** confirmed the presence of an aldehyde group based on the direct correlation from a downfield proton at δ_H_ 10.05 (H-1) to a carbonyl carbon (δ_C_ 194.5, C-1). The HMBC spectrum of **5** confirmed the positions of its aldehyde moiety, aliphatic side chain, and methyl group on the aromatic ring at C-1a, C-3, and C-6 supported by key correlations (Figure 4) from H-1 to C-1a (δ_C_ 113.8) and C-2 (δ_C_ 164.8); from H_2_-1′ and H_2_-2′ to C-3 (δ_C_ 114.4); from H_3_-7 to C-1a, C-5 (δ_C_ 111.0) and C-6 (δ_C_ 143.3), respectively. In addition, the ROESY spectrum of **5** exhibited key NOE correlations between aldehyde proton (H-1) and aromatic methyl group (H_3_-7). Based on the obtained results, compound **5** was deduced to be a new phenolic derivative structurally related to hericenes **A–C**,^22^ bearing a terminal carboxylic acid group at the end of its aliphatic side chain as deduced from its HMBC spectrum that revealed key correlations from a methylene group (δ_H_ 2.98, H_2_-5′) and an olefinic proton (δ_H_ 5.28, H-4′) to a carboxylic acid carbon (δ_C_ 176.6, C-6′). Compound **5** was given a trivial name hericioic acid E.

**Table 2:** ^1^H and ^13^C NMR Data of **5–8**

	**5**		**6**		**7**		**8**	
Pos.	δ_C_,^a^^,^^c^ type	δ_H_^b^ (multi, *J* [Hz])	δ_C_,^a^^,^^c^ type	δ_H_^b^ (multi, *J* [Hz])	δ_C_,^a^^,^^c^ type	δ_H_^b^ (multi, *J* [Hz])	δ_C_,^a^^,^^c^ type	δ_H_^b^ (multi, *J* [Hz])
1	194.5, CO	10.04 (br s, 1H)	197.1, CO	10.43 (br s, 1H)	175.4, CO		146.1, C	8.14 (d, 1.3, 1H)
1a	113.8, C		113.2, C		103.9, C		123.6, C	
2	164.8, C		163.2, C		164.8, C		182.8, CO	
3	114.4, C		120.2, C		115.1, C		127.0, C	
4	165.2, C		164.9, C		161.6, C		159.8, C	
5	111.0, CH	6.22 (s, 1H)	111.9, CH	7.00 (s, 1H)	107.4, CH	6.62 (s, 1H)	177.7, CO	
5a	143.3, C		135.5, C		144.9, C		121.5, C	
6	18.1, CH_3_	2.47 (s, 3H)	169.3, CO		64.8, CH_2_	4.79 (s, 2H)	147.2, C	8.28 (d, 1.3, 1H)
1′	21.8, CH_2_	2.69 (t, 8.0, 2H)	22.3, CH_2_	2.78 (t, 8.0, 2H)	22.7, CH_2_	2.73 (t, 8.0, 2H)	22.7, CH_2_	2.65 (t, 7.7, 2H)
2′	39.5, CH_2_	2.19 (t, 8.0, 2H)	39.0, CH_2_	2.23 (t, 8.0, 2H)	39.5, CH_2_	2.20 (t, 8.0, 2H)	38.7, CH_2_	2.20 (t, 7.7, 2H)
3′	140.1, C		140.0, C		140.6, C		139.5, C	
4′	117.5, CH	5.28 (td, 7.2, 1.4, 1H)	117.6, CH	5.28 (td, 7.0, 1.5, 1H)	117.1, CH	5.30 (td, 7.2, 1.4, 1H)	118.1, CH	5.27 (td, 7.1, 1.4, 1H)
5′	34.6, CH_2_	2.98 (d, 7.2, 2H)	34.4, CH_2_	2.99 (d, 7.0, 2H)	34.4, CH_2_	3.00 (d, 7.2, 2H)	34.4, CH_2_	2.97 (d, 7.1, 2H)
6′	176.6, CO		176.3, CO		176.5, CO		176.2, CO	
7′	16.3, CH_3_	1.73 (d, 1.4, 3H)	16.3, CH_3_	1.74 (d, 1.5, 3H)	16.4, CH_3_	1.74 (d, 1.4, 3H)	16.2, CH_3_	1.72 (d, 1.4, 3H)

a Measured in methanol-*d*_4_ at 175 MHz.

b Measured in methanol-*d*_4_ at 700 MHz.

c Assigned based on HMBC and HSQC spectra.

Compound **6** was obtained as a brown amorphous solid and its molecular formula was determined to be C_15_H_16_O_7_ supported by the pseudomolecular ion peak in HRESIMS at *m/z* 309.0973 [M + H]^+^ (calculated 309.0969) indicating its possession of eight degrees of unsaturation compared to seven in **5**. The ^13^C NMR data of **6** (Table 2) provided an explanation to this structural difference compared to hericioic acid E (**5**) by illustrating the presence of one carboxylic acid moiety at δ_C_ 169.3 replacing the aromatic methyl group at δ_H_ 2.47 (d, *J* = 0.7 Hz, H_3_-7; δ_C_ 18.1) in **5**. To further confirm the position of the new carboxylic acid moiety in **6**, its HMBC spectrum (Figure 4) was measured that displayed a clear correlation from an aromatic proton at δ_H_ 7.00 (H-5) to the carboxylic acid group indicating its binding at C-6. Apart from that difference, the 1D (^1^H and ^13^C) and 2D (^1^H–^1^H COSY, HMBC, HSQC, and ROESY) NMR spectral data of **5** and **6** were comparable, supporting its identity as a new derivative named hericioic acid **F**.

The molecular formula of compound **7**, purified as a brown oil, was established to be C_15_H_18_O_7_ according to the pseudomolecular ion peak at *m/z* 311.1127 [M + H]^+^ (calculated 311.1125) indicating the presence of seven degrees of unsaturation. By comparing the molecular formulas and the degrees of unsaturation of **6** and **7**, the latter was noticed to have two additional protons and one less degree of unsaturation that can be attributed to a saturated double bond. The ^1^H and ^13^C NMR spectral data (Table 2) clearly explained this structural difference by showing the presence of a hydroxymethylene group at δ_H_ 4.79 (s, H_2_-7; δ_C_ 64.8) in **7** instead of an aldehyde group at δ_H_ 10.43 (H-1; δ_C_ 197.1) in **6**. To further confirm the location of the introduced hydroxymethylene moiety, HMBC and HSQC spectra of **7** (Figure 4) were recorded that revealed key correlations from the hydroxymethylene group to four quaternary carbons at δ_C_ 164.8 (C-2), δ_C_ 161.6 (C-4), δ_C_ 144.9 (C-6), and δ_C_ 103.9 (C-1a) together with a methine aromatic carbon at δ_C_ 107.4 (C-5) confirming its position at C-6. An additional long range (ω) correlation can be distinguished in HMBC from an aromatic proton H-5 at δ_H_ 6.62 to a carboxylic acid carbon (δ_C_ 175.4, C-1). In addition, 1D (^1^H and ^13^C) and 2D (^1^H–^1^H COSY, HMBC, HSQC, and ROESY) NMR spectral data of **7** undoubtedly confirmed the existence of the common aliphatic side chain ending with a free carboxylic acid moiety as in hericioic acids **E** (**5**) and **F** (**6)** and similarly bound at C-3. Based on the obtained results, compound **7** was identified as a new derivative and named as hericioic acid **G**.

Compound **8** was obtained as an off-white amorphous solid with a characteristic UV spectrum revealing absorption peaks (λ_max_) at 201, 249 and 348 nm. The molecular formula of **8** was determined to be C_15_H_14_O_6_ as shown by its HRESIMS that revealed pseudomolecular ion peaks at *m/z* 291.0858 [M + H]^+^ (calculated 291.0863) and at *m/z* 313.0681 [M + Na]^+^ (calculated 313.0683) indicating its inclusion of nine degrees of unsaturation.

The ^13^C NMR and HSQC spectra of **8** (Table 2) disclosed 15 carbon peaks differentiated into eight quaternary carbons comprising 3 carbonyls (δ_C_ 182.8, 177.7, 176.2), 5 olefinic carbon atoms with one oxygenated (δ_C_ 159.8, 139.5, 127.0, 123.6, 121.5), and 3 tertiary methines as 2 downfield carbon atoms at δ_C_ 147.2 and δ_C_ 146.1 and a third olefinic methine carbon at δ_C_ 118.1 that altogether account for 7 degrees of unsaturation suggesting compound **8** to have a bicyclic structure. In addition, the ^13^C NMR data of **8** also showed the presence of three methylenes (δ_C_ 38.7, δ_C_ 34.4, δ_C_ 22.7) and one olefinic methyl carbon (δ_C_ 16.2). The ^1^H–^1^H COSY and HSQC spectra of **8** (Table 2) revealed three main spin systems as follows, namely, between two downfield aromatic protons at δ_H_ 8.14 (H-1) and δ_H_ 8.28 (H-6); between two aliphatic methylene groups at δ_H_ 2.65 (dd, *J* = 6.8, 8.6 Hz, H_2_-1′) and at δ_H_ 2.20 (t, *J* = 7.7 Hz, H_2_-2′); from an olefinic proton at δ_H_ 5.27 (dd, *J* = 1.3, 7.1 Hz, H-4′) showing vicinal and allylic coupling to a methylene group at δ_H_ 2.97 (d, *J* = 7.1 Hz, H_2_-5′) and a methyl peak at δ_H_ 1.72 (d, *J* = 1.3 Hz, H_3_-7′), respectively. The HMBC spectrum of **8** showed key correlations (Figure 4) from the two downfield protons at H-1 and H-6 to two carbonyl carbon atoms at δ_C_ 182.8 (C-2) and δ_C_ 177.7 (C-5) indicating the chemical structure of **8** (Figure 1) to be containing an isobenzofuran-2,5-quinone moiety similar to the previously reported metabolites^24^ along with having an aliphatic side chain ending with a free carboxylic acid as in hericioic acids **E–G** (**5**–**7**). Further proofs for the position of the side chain were provided by HMBC spectrum that revealed key correlations from H_2_-1′ to C-2, C-3 (δ_C_ 127.0) and C-4 (δ_C_ 159.8) indicating its presence as a side chain at C-3. The presence of a terminal free carboxylic acid moiety in **8** was also confirmed by HMBC spectrum that exhibited key correlations from a methylene group (δ_H_ 2.97, H_2_-5′), an olefinic proton (δ_H_ 5.27, H-4′), and an olefinic methyl group (δ_H_ 1.72, H_3_-7′) to a carboxylic acid carbon (δ_C_ 176.2, C-6′). In conclusion, compound **8** was determined to be a new isofuran-2,5-quinone derivative and was trivially named as hericiofuranoic acid.

### 3.1 Hericioic Acid **A** (**1**)

Off-white amorphous solid; [α]_D_^20^ + 22° (*c* 0.1, DMSO); CD (DMSO): λ_max_ (Δε) 192 (2.2), 231 (−2.5) nm; UV/vis (DMSO): λ_max_ 218, 257, 297 nm; IR (neat) ν_max_: 3273, 2940, 1646, 1124, 1012, 950, 772 cm^–1^; NMR data (^1^H NMR: 600 MHz, ^13^C NMR: 150 MHz, (CD_3_)_2_SO) see Table 1; HR-(+)ESIMS: *m/z* 262.1067 [M–H_2_O + H]^+^ (calcd 262.1072 for C_14_H_16_NO_4_^+^), 280.1176 [M + H]^+^ (calcd 280.1179 for C_14_H_18_NO_5_^+^), 302.0994 [M + Na]^+^ (calcd 302.0999 for C_14_H_17_NNaO_5_^+^); *t*_R_ = 5.42 min (HR-LC-ESIMS). C_14_H_17_NO_5_ (279.29 g/mol).

### 3.2 Hericioic Acid **B** (**2**)

White amorphous solid; UV/vis (DMSO): λ_max_ 221, 256, 300 nm; IR (neat) ν_max_: 3304, 2943, 1675, 1463, 1167, 768 cm^–1^; NMR data (^1^H NMR: 600 MHz, ^13^C NMR: 150 MHz, (CD_3_)_2_SO) see Table 1; HR-(+)ESIMS: *m/z* 274.1069 [M–H_2_O + H]^+^ (calcd 274.1074 for C_15_H_16_NO_4_^+^), 292.1179 [M + H]^+^ (calcd 292.1179 for C_15_H_18_NO_5_^+^), 314.0994 [M + Na]^+^ (calcd 314.0999 for C_15_H_17_NNaO_5_^+^), 583.2287 [2M + H]^+^ (calcd 583.2286 for C_30_H_35_N_2_O_10_^+^), 605.2099 [2M + Na]^+^ (calcd 605.2106 for C_30_H_34_N_2_NaO_10_^+^); *t*_R_ = 5.07 min (HR-LC-ESIMS). C_15_H_17_NO_5_ (291.30 g/mol).

### 3.3 Hericioic Acid **C** (**3**)

White amorphous solid; UV/vis (DMSO): λ_max_ 219, 258, 296 nm; IR (neat) ν_max_: 3251, 2942, 1664, 1471, 1166, 1115, 771 cm^–1^; NMR data (^1^H NMR: 600 MHz, ^13^C NMR: 150 MHz, (CD_3_)_2_SO) see Table 1; HR-(+)ESIMS: *m/z* 306.1334 [M + H]^+^ (calcd 306.1336 for C_16_H_20_NO_5_^+^), 328.1153 [M + Na]^+^ (calcd 328.1155 for C_16_H_19_NNaO_5_^+^); *t*_R_ = 6.36 min (HR-LC-ESIMS). C_16_H_19_NO_5_ (305.33 g/mol).

### 3.4 Hericioic Acid **D** (**4**)

Yellow amorphous solid; UV/vis (DMSO): λ_max_ 220, 257, 300 nm; IR (neat) ν_max_: 3251, 2934, 1684, 1471, 1163, 1109, 770 cm^–1^; NMR data (^1^H NMR: 600 MHz, ^13^C NMR: 150 MHz, (CD_3_)_2_SO) see Table 1; HR-(+)ESIMS: *m/z* 328.1537 [M–H_2_O + H]^+^ (calcd 328.1543 for C_19_H_22_NO_4_^+^), 346.1649 [M + H]^+^ (calcd 346.1649 for C_19_H_24_NO_5_^+^), 368.1463 [M + Na]^+^ (calcd 368.1468 for C_19_H_23_NNaO_5_^+^); *t*_R_ = 8.40 min (HR-LC-ESIMS). C_19_H_23_NO_5_ (345.40 g/mol).

### 3.5 Hericioic Acid **E** (**5**)

Brown oil; UV/vis (MeOH): λ_max_ 200, 226, 300 nm; NMR data (^1^H NMR: 700 MHz, ^13^C NMR: 175 MHz, CD_3_OD) see Table 2; HR-(+)ESIMS: *m/z* 261.1122 [M–H_2_O + H]^+^ (calcd 261.1121 for C_15_H_17_O_4_^+^), 279.1228 [M + H]^+^ (calcd 279.1227 for C_15_H_19_O_5_^+^), 301.1048 [M + Na]^+^ (calcd 301.1046 for C_15_H_18_NaO_5_); *t*_R_ = 8.23 min (HR-LC-ESIMS). C_15_H_18_O_5_ (278.30 g/mol).

### 3.6 Hericioic Acid **F** (**6**)

Brown amorphous solid; UV/vis (MeOH): λ_max_ 200, 234, 299 nm; IR (neat) ν_max_: 2920, 1704, 1609, 1236, 1101, 1005, 947, 773 cm^–1^; NMR data (^1^H NMR: 700 MHz, ^13^C NMR: 175 MHz, CD_3_OD) see Table 2; HR-(+)ESIMS: *m/z* 291.0867 [M–H_2_O + H]^+^ (calcd 291.0863 for C_15_H_15_O_6_^+^), 309.0973 [M + H]^+^ (calcd 309.0969 for C_15_H_17_O_7_^+^), 331.0791 [M + Na]^+^ (calcd 331.0788 for C_15_H_16_NaO_7_); *t*_R_ = 7.22 min (HR-LC-ESIMS). C_15_H_16_O_7_ (308.29 g/mol).

### 3.7 Hericioic Acid **G** (**7**)

Brown oil; UV/vis (MeOH): λ_max_ 229, 269, 304 nm; NMR data (^1^H NMR: 700 MHz, ^13^C NMR: 175 MHz, CD_3_OD) see Table 2; HR-(+)ESIMS: *m/z* 293.1022 [M–H_2_O + H]^+^ (calcd 293.1020 for C_15_H_17_O_6_^+^), 311.1127 [M + H]^+^ (calcd 311.1125 for C_15_H_19_O_7_^+^), 333.0945 [M + Na]^+^ (calcd 333.0945 for C_15_H_18_NaO_7_); *t*_R_ = 5.99 min (HR-LC-ESIMS). C_15_H_18_O_7_ (310.30 g/mol).

### 3.8 Hericiofuranoic Acid (**8**)

Off-white amorphous solid; UV/vis (MeOH): λ_max_ 201, 249, 348 nm; IR (neat) ν_max_: 2934, 1710, 1673, 1645, 1552, 1185, 1009, 877 cm^–1^; NMR data (^1^H NMR: 700 MHz, ^13^C NMR: 175 MHz, CD_3_OD) see Table 2; HR-(+)ESIMS: *m/z* 273.0756 [M–H_2_O + H]^+^ (calcd 273.0757 for C_15_H_13_O_5_^+^), 291.0858 [M + H]^+^ (calcd 291.0863 for C_15_H_15_O_6_^+^), 313.0681 [M + Na]^+^ (calcd 313.0683 for C_15_H_14_NaO_6_; *t*_R_ = 6.72 min (HR-LC-ESIMS). C_15_H_14_O_6_ (290.27 g/mol).

Hericioic acids **A–G** (**1**–**7**) and hericiofuranoic acid (**8**) were evaluated for their cytotoxic effects based on the established protocol.^14^ None of the tested compounds exhibited significant growth inhibition against sensitive mouse fibroblast cells (L929) or human endocervical adenocarcinoma (KB 3.1). Hence, further tests on the other cell lines were not performed. Comparable observations had been made on similar compounds as previously reported.^6,25^

**Figure 5:**
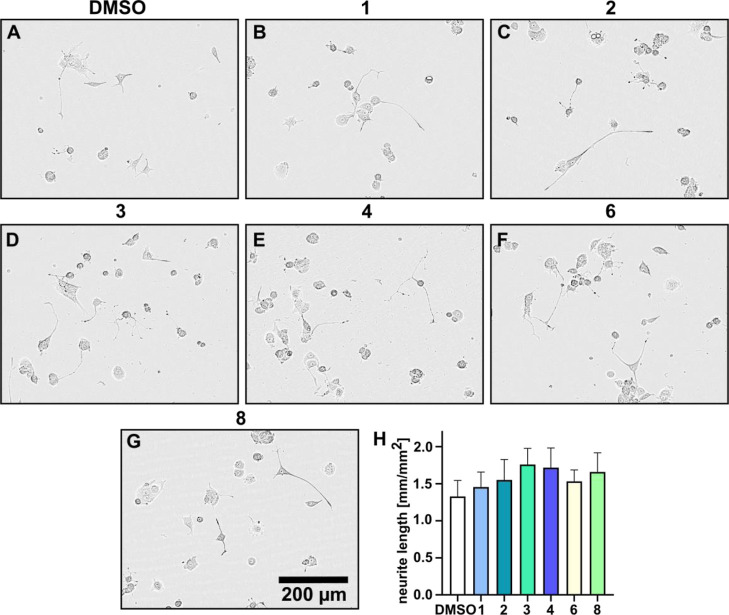
PC-12 cells were treated with (A) DMSO, (B–F) 1 mg/mL hericioic acids (**1**–**4, 6**), and (G) hericiofuranoic acid (**8**) supplemented with 5 ng/mL NGF. Phase contrast images show neurite outgrowth after 48 h of treatment. (H) Bar graph displays the mean neurite length ± SEM, from five independent experiments.

Differentiation and neurite outgrowth of PC-12 cells is a commonly used model to study neurotrophic effects of drugs and fungal metabolites.^16^ Therefore, we investigated the neurotrophic potential of hericioic acids (**1**–**4**, **6**) and hericiofuranoic acid (**8**), supplemented with 5 ng/mL NGF, by measuring the mean neurite length after 48 h of treatment (Figure 5). Interestingly, we noticed that the treatment with compounds (**3**, **4,** or **8**) promoted a stronger neurite outgrowth in PC-12 cells as compared to the DMSO control. The other compounds had similar effects, albeit moderately. Hence, we provide the first evidence on the neurotrophic potential of the newly described isoindolinone and benzofuranone derivatives; hericioic acids (**1**–**4**, **6**) and hericiofuranoic acid (**8**).

Our findings clearly support the medicinal value reports of the genus *Hericium* and expands the chemistry of its compounds. The visualized neurite outgrowth stimulatory effects of the metabolites corroborates with neurotrophic effects of similar structures from *Hericium coralloides* and *H. erinaceus* basidiomes^6,9,25^ in addition to the rice culture of the latter.^26,27^ This gives a glimpse into the structure–activity relationships (SARs) of the compounds with a similar core structure. The substitution on the nitrogen, aromatic hydroxyl groups, as well as the stereochemistry and the length of the aliphatic side chain influenced the α-glucosidase inhibitory activity of isoindolinone derivatives.^25^ In this study, the obtained results from neurotrophic assay revealed a pattern similar to that previously reported^25^ where hericioic acid A (**1**) with the shortest side chain exhibited the least activity. Nonetheless, compounds (**2**–**8**) had no significant differences in their activities to conclusively deduce their SARs. It also remains worthwhile to note that related compounds of isoindolinone and benzofuranone scaffolds were so far only reported from the basidiomata^6,9^ and rice cultures^25^ of *Hericium* species, while the submerged cultures are rich in cyathanes.^7,8^ In order to assure sustainable access to the isolated compounds and finding out more derivatives to assess the SARs, further solid-state cultivations should be further exploited in the future.

## Supporting Information Available

All 1D (^1^H/^13^C) and 2D (^1^H–^1^H COSY, HMBC, HSQC, and ROESY) spectra in addition to all HPLC-ESI-MS and HRESIMS of **1–8** (PDF)
