# Supplementary material for: Hericioic Acids A–G and Hericiofuranoic Acid; Neurotrophic Agents from Cultures of the European Mushroom Hericium flagellum
Source: J Agric Food Chem. 2023 Jul 13;71(29):11094–103. doi: 10.1021/acs.jafc.3c02897 (PMC10375585; doi:10.1021/acs.jafc.3c02897)
Supplement: Supplementary file 1 — jf3c02897_si_001.pdf [file jf3c02897_si_001.pdf]

Supplementary Materials for

Hericioic acids A-G and Hericiofuranoic acid; Neurotrophic agents from cultures of the European Mushroom *Hericium flagellum*

Winnie Chemutai Sum[1,2], Sherif Saeed Ebada[1,3], Marco Kirchenwitz[4], Harald Kellner[5], Mahmoud A. A. Ibrahim[6,7], Theresia E. B. Stradal[4], Josphat Clement Matasyoh[8] and Marc Stadler[1,2]*

[1] Department of Microbial Drugs, Helmholtz Centre for Infection Research GmbH (HZI), Inhoffenstraße 7, 38124 Braunschweig, Germany; winnie.sumchemutai@helmholtz-hzi.de; sherif.elsayed@helmholtz-hzi.de

[2] Institute of Microbiology, Technische Universität Braunschweig, Spielmannstraße 7, 38106 Braunschweig, Germany

[3] Department of Pharmacognosy, Faculty of Pharmacy, Ain Shams University, 11566 Cairo, Egypt; sherif_elsayed@pharma.asu.edu.eg

[4] Department of Cell Biology, Helmholtz Centre for Infection Research, Inhoffenstrasse 7, 38124 Braunschweig, Germany; marco.kirchenwitz@helmholtz-hzi.de; theresia.stradal@helmholtz-hzi.de

[5] Department of Bio- and Environmental Sciences, Technische Universität Dresden-International Institute Zittau, Markt 23, 02763 Zittau, Germany; harald.kellner@tu-dresden.de

[6] Computational Chemistry Laboratory, Chemistry Department, Faculty of Science, Minia University, Minia 61519, Egypt; m.ibrahim@compchem.net

[7] School of Health Sciences, University of KwaZulu-Natal, Westville, Durban 4000, South Africa

[8] Department of Chemistry, Egerton University, P.O. Box 536, 20115, Njoro, Kenya; jmatasyoh@egerton.ac.ke

* Correspondence: Marc.Stadler@helmholtz-hzi.de; Tel.: +49-531-6181-4240

ABSTRACT

Neurodegenerative diseases (NDDs) are currently posing huge social, economic and healthcare burdens among the aged populations worldwide with few and only palliative treatment alternatives available. Natural products continue to be a source for a vast array of potent neurotrophic molecules that could be considered as drug design starting points. The present study reports eight new isoindolinone and benzofuranone derivatives, for which we propose the trivial names, hericioic acids A-G (**1**-**7**) and hericiofuranoic acid (**8**), which were isolated from a solid culture (using rice as substrate) of the rare European edible mushroom *Hericium flagellum*. The chemical structures of these compounds were determined based on extensive 1D and 2D NMR spectroscopy along with HRESIMS analyses. The isolated compounds were assessed for their neurotrophic activity in rat pheochromocytoma cells (PC-12) to promote neurite outgrowth on 5 ng NGF supplementation; all the compounds increased neurite outgrowths, with compounds **3**, **4** and **8** exhibiting the strongest effects.

Keywords: Basidiomycota; Hericiaceae, isoindolinone; benzofuranone; neurotrophic

Contents of Supporting Information

#	Contents	Page
**1**	Figure S1. HPLC chromatogram and LRESIMS spectrum of **1**.	**S5**
**2**	Figure S2. HPLC chromatogram and HRESIMS spectrum of **1**.	**S6**
**3**	Figure S3. ^{1}H NMR spectrum of **1** in DMSO-d_6 at 600 MHz.	**S7**
**4**	Figure S4. ^{13}C NMR spectrum of **1** in DMSO-d_6 at 150 MHz.	**S8**
**5**	Figure S5. ^{1}H-^{1}H COSY spectrum of **1** in DMSO-d_6 at 600 MHz.	**S9**
**6**	Figure S6. HMBC spectrum of **1** in DMSO-d_6 at 600 MHz.	**S10**
**7**	Figure S7. HSQC spectrum of **1** in DMSO-d_6 at 600 MHz.	**S11**
**8**	Figure S8. ROESY spectrum of **1** in DMSO-d_6 at 600 MHz.	**S12**
**9**	Figure S9. Experimental and calculated ECD spectrum of **1**.	**S13**
**10**	Figure S10. HPLC chromatogram and LRESIMS spectrum of **2**.	**S14**
**11**	Figure S11. HPLC chromatogram and HRESIMS spectrum of **2**.	**S15**
**12**	Figure S12. ^{1}H NMR spectrum of **2** in DMSO-d_6 at 600 MHz.	**S16**
**13**	Figure S13. ^{13}C NMR spectrum of **2** in DMSO-d_6 at 150 MHz.	**S17**
**14**	Figure S14. ^{1}H-^{1}H COSY spectrum of **2** in DMSO-d_6 at 600 MHz.	**S18**
**15**	Figure S15. HMBC spectrum of **2** in DMSO-d_6 at 600 MHz.	**S19**
**16**	Figure S16. HSQC spectrum of **2** in DMSO-d_6 at 600 MHz.	**S20**
**17**	Figure S17. ROESY spectrum of **2** in DMSO-d_6 at 600 MHz.	**S21**
**18**	Figure S18. HPLC chromatogram and LRESIMS spectrum of **3**.	**S22**
**19**	Figure S19. HPLC chromatogram and HRESIMS spectrum of **3**.	**S23**
**20**	Figure S20. ^{1}H NMR spectrum of **3** in DMSO-d_6 at 600 MHz.	**S24**
**21**	Figure S21. ^{13}C NMR spectrum of **3** in DMSO-d_6 at 150 MHz.	**S25**
**22**	Figure S22. ^{1}H-^{1}H COSY spectrum of **3** in DMSO-d_6 at 600 MHz.	**S26**
**23**	Figure S23. HMBC spectrum of **3** in DMSO-d_6 at 600 MHz.	**S27**
**24**	Figure S24. HSQC spectrum of **3** in DMSO-d_6 at 600 MHz.	**S28**
**25**	Figure S25. ROESY spectrum of **3** in DMSO-d_6 at 600 MHz.	**S29**
**26**	Figure S26. HPLC chromatogram and LRESIMS spectrum of **4**.	**S30**
**27**	Figure S27. HPLC chromatogram and HRESIMS spectrum of **4**.	**S31**
**28**	Figure S28. ^{1}H NMR spectrum of **4** in DMSO-d_6 at 600 MHz.	**S32**
**29**	Figure S29. ^{13}C NMR spectrum of **4** in DMSO-d_6 at 150 MHz.	**S33**
**30**	Figure S30. ^{1}H-^{1}H COSY spectrum of **4** in DMSO-d_6 at 600 MHz.	**S34**
**31**	Figure S31. HMBC spectrum of **4** in DMSO-d_6 at 600 MHz.	**S35**
**32**	Figure S32. HSQC spectrum of **4** in DMSO-d_6 at 600 MHz.	**S36**
**33**	Figure S33. ROESY spectrum of **4** in DMSO-d_6 at 600 MHz.	**S37**
**34**	Figure S34. HPLC chromatogram and LRESIMS spectrum of **5**.	**S38**
**35**	Figure S35. HPLC chromatogram and HRESIMS spectrum of **5**.	**S39**
**36**	Figure S36. ^{1}H NMR spectrum of **5** in methanol-d_4 at 700 MHz.	**S40**
**37**	Figure S37. ^{13}C NMR spectrum of **5** in methanol-d_4 at 175 MHz.	**S41**
**38**	Figure S38. ^{1}H-^{1}H COSY spectrum of **5** in methanol-d_4 at 700 MHz.	**S42**
**39**	Figure S39. HMBC spectrum of **5** in methanol-d_4 at 700 MHz.	**S43**
**40**	Figure S40. HSQC spectrum of **5** in methanol-d_4 at 700 MHz.	**S44**
**41**	Figure S41. ROESY spectrum of **5** in methanol-d_4 at 700 MHz.	**S45**
**42**	Figure S42. HPLC chromatogram and LRESIMS spectrum of **6**.	**S46**
**43**	Figure S43. HPLC chromatogram and HRESIMS spectrum of **6**.	**S47**
**44**	Figure S44. ^{1}H NMR spectrum of **6** in methanol-d_4 at 700 MHz.	**S48**
**45**	Figure S45. ^{13}C NMR spectrum of **6** in methanol-d_4 at 175 MHz.	**S49**
**46**	Figure S46. ^{1}H-^{1}H COSY spectrum of **6** in methanol-d_4 at 700 MHz.	**S50**
**47**	Figure S47. HMBC spectrum of **6** in methanol-d_4 at 700 MHz.	**S51**
**48**	Figure S48. HSQC spectrum of **6** in methanol-d_4 at 700 MHz.	**S52**

**49**	Figure S49. ROESY spectrum of **6** in methanol-d_4 at 700 MHz.	**S53**
**50**	Figure S50. HPLC chromatogram and LRESIMS spectrum of **7**.	**S54**
**51**	Figure S51. HPLC chromatogram and HRESIMS spectrum of **7**.	**S55**
**52**	Figure S52. ^{1}H NMR spectrum of **7** in methanol-d_4 at 700 MHz.	**S56**
**53**	Figure S53. ^{13}C NMR spectrum of **7** in methanol-d_4 at 175 MHz.	**S57**
**54**	Figure S54. ^{1}H-^{1}H COSY spectrum of **7** in methanol-d_4 at 700 MHz.	**S58**
**55**	Figure S55. HMBC spectrum of **7** in methanol-d_4 at 700 MHz.	**S59**
**56**	Figure S56. HSQC spectrum of **7** in methanol-d_4 at 700 MHz.	**S60**
**57**	Figure S57. ROESY spectrum of **7** in methanol-d_4 at 700 MHz.	**S61**
**58**	Figure S58. HPLC chromatogram and LRESIMS spectrum of **8**.	**S62**
**59**	Figure S59. HPLC chromatogram and HRESIMS spectrum of **8**.	**S63**
**60**	Figure S60. ^{1}H NMR spectrum of **8** in methanol-d_4 at 700 MHz.	**S64**
**61**	Figure S61. ^{13}C NMR spectrum of **8** in methanol-d_4 at 175 MHz.	**S65**
**62**	Figure S62. ^{1}H-^{1}H COSY spectrum of **8** in methanol-d_4 at 700 MHz.	**S66**
**63**	Figure S63. HMBC spectrum of **8** in methanol-d_4 at 700 MHz.	**S67**
**64**	Figure S64. HSQC spectrum of **8** in methanol-d_4 at 700 MHz.	**S68**
**65**	Figure S65. ROESY spectrum of **8** in methanol-d_4 at 700 MHz.	**S69**
**66**	Figure S66. PC-12 cells treated with DMSO, hericioic acids (**1**−**4**, **6**) and hericiofuranoic acid (**8**).	**S70**

Generic Display Report

Analysis Info

		Acquisition Date	13.04.2022 20:29:10
Analysis Name	S:\PEOPLE\sel22_Sherif Elsayed\Hericium\Amazon\Hericium fl. Rice F8F2_BD2_01_10893.d		
Method	Screening_Set-05iso-uv210_10893.m	Operator	lab
Sample Name	Hericium fl. Rice F8F2	Instrument	amaZon speed
Comment			

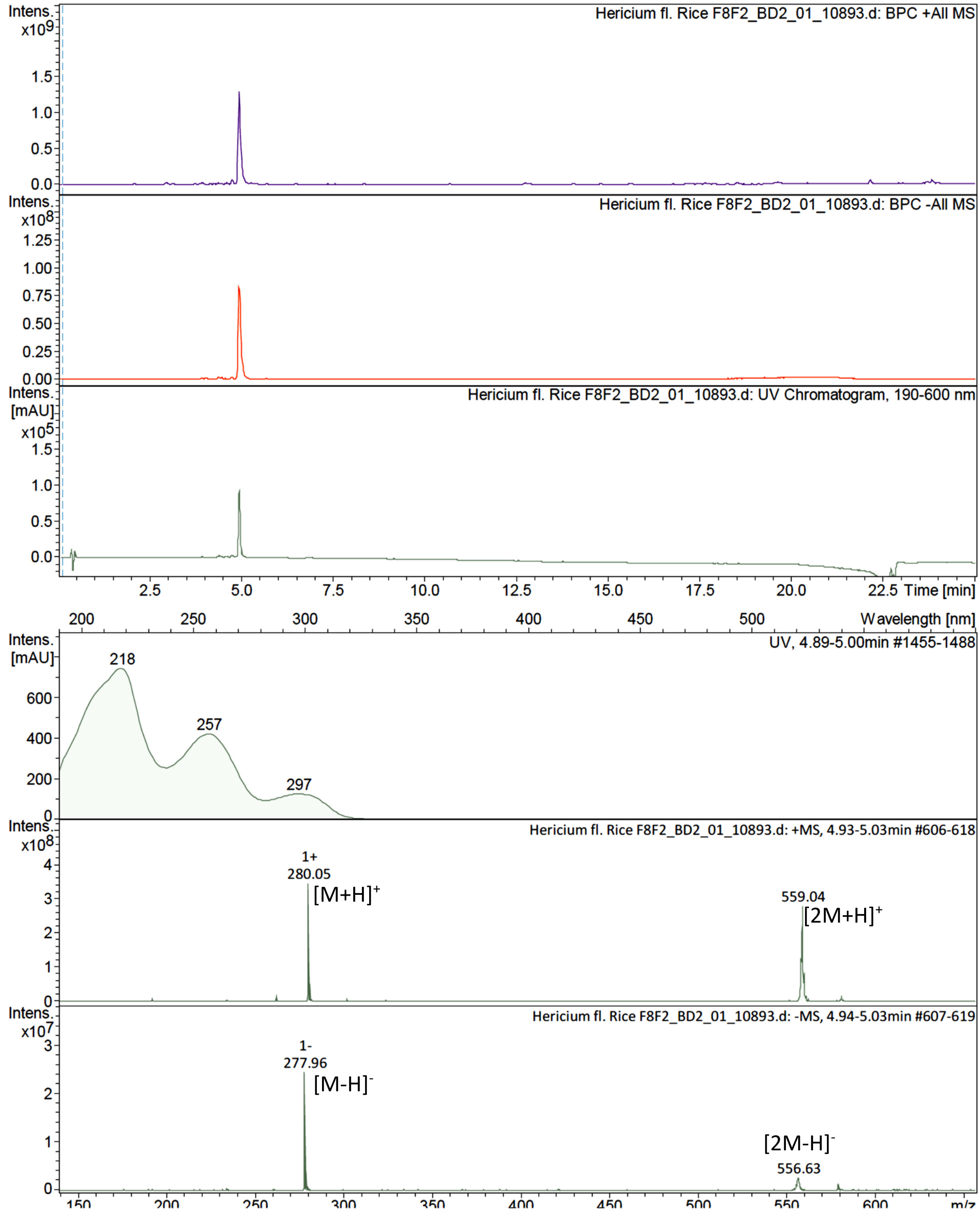

Bruker Compass DataAnalysis 4.4 printed: 26.03.2023 14:19:01 by: sel22 Page 1 of 1

Figure S1. HPLC chromatogram and LRESIMS spectrum of **1**.

Generic Display Report

**Analysis Info**		Acquisition Date	25.04.2022 10:16:07
Analysis Name	S:\PEOPLE\sel22_Sherif Elsayed\Hericium\Hericium fl MaXis\(279)		
Method	pos_säure_10000_screening_ms_100_2500_line.m	Operator	ate06
Sample Name	Hericium Rice F8F2	Instrument	maXis
Comment	Screening01<br>Waters Acquity UPLC BEH C_18 1,7um 2.1x50mm		

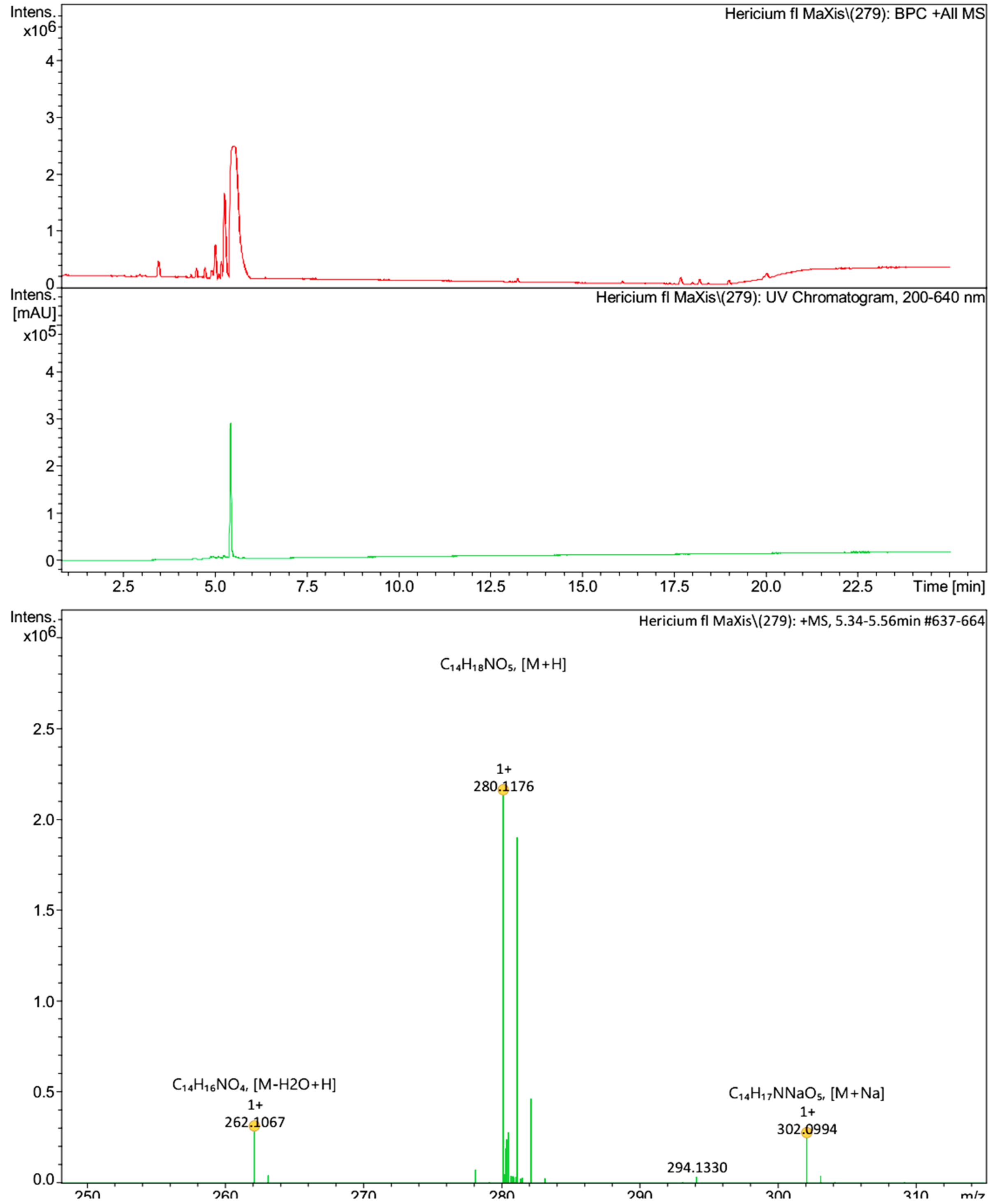

Bruker Compass DataAnalysis 4.4 printed: 01.04.2023 15:45:52 by: sel22 Page 1 of 1

Figure S2. HPLC chromatogram and HRESIMS spectrum of **1**.

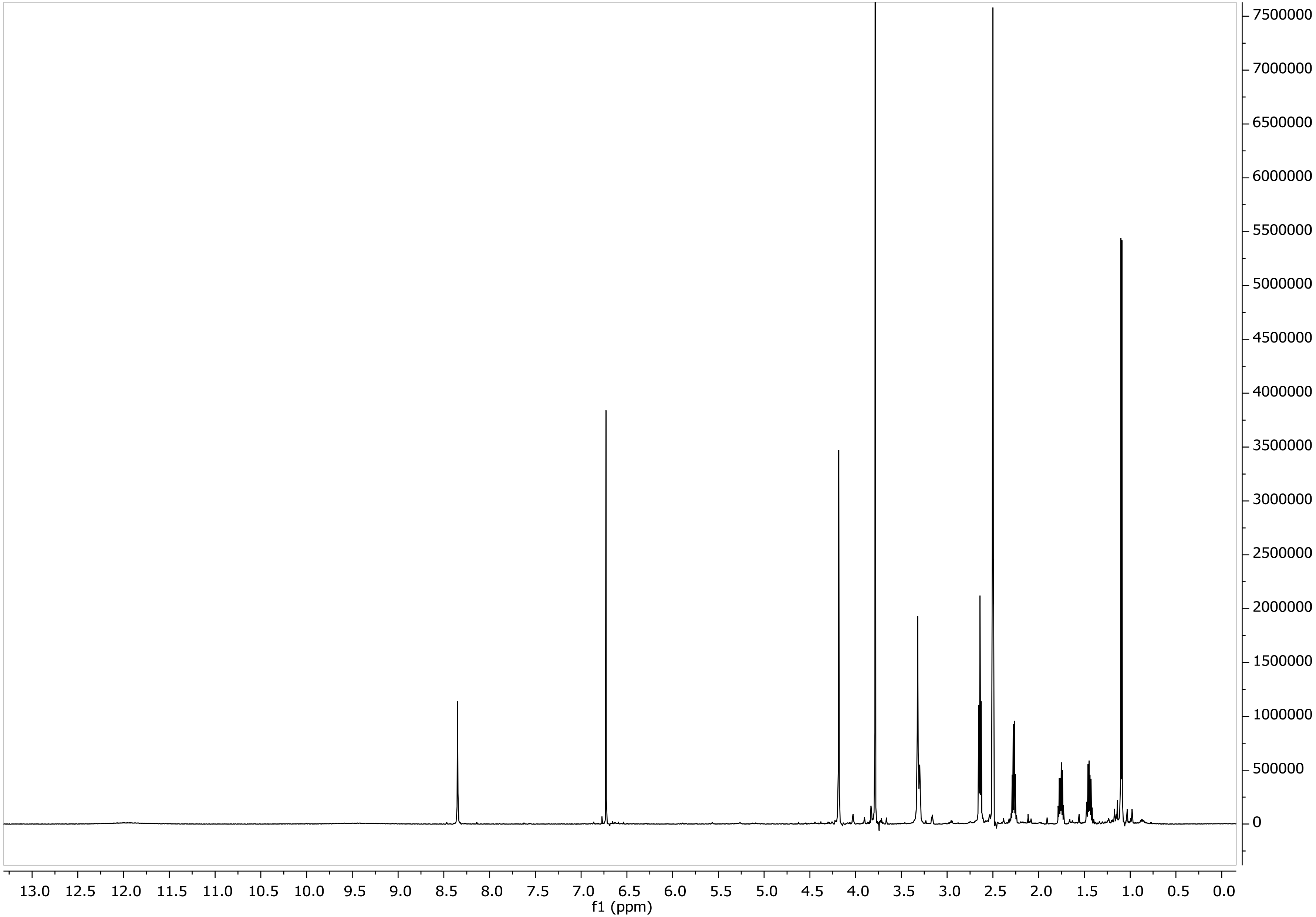

Figure S3. ^{1}H NMR spectrum of **1** in DMSO-d_6 at 600 MHz.

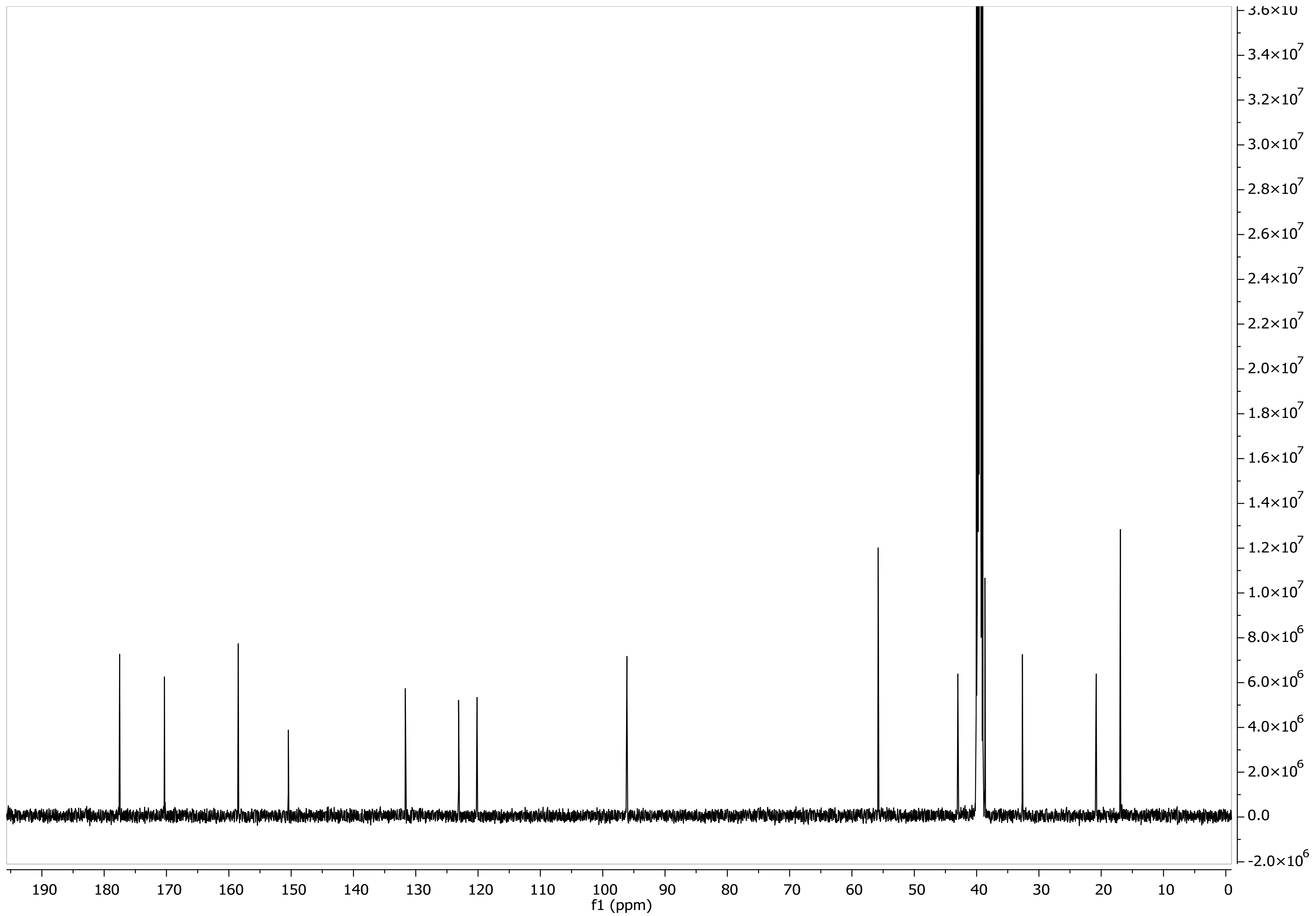

Figure S4. ^{13}C NMR spectrum of **1** in DMSO-d_6 at 150 MHz.

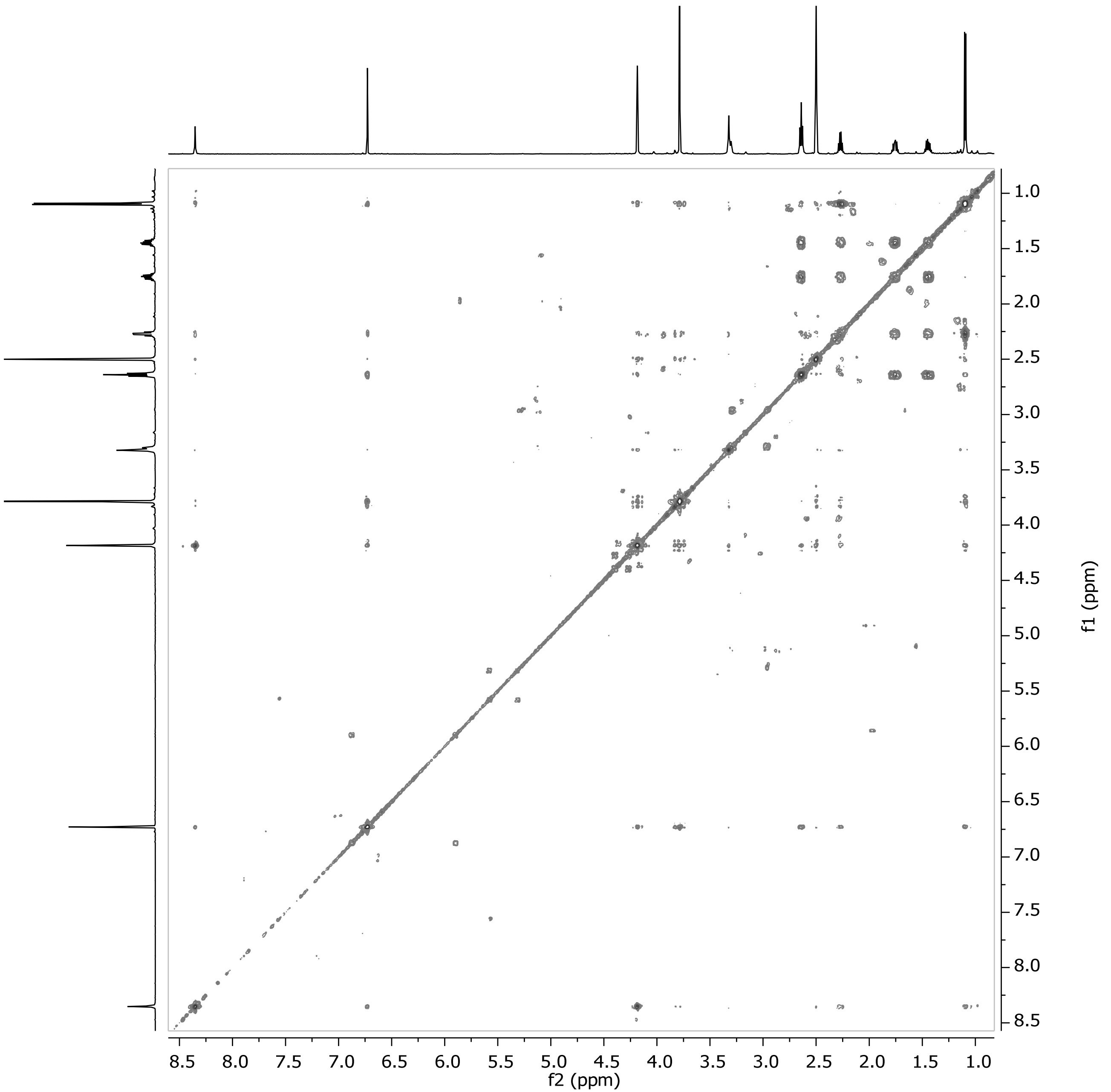

Figure S5. ^{1}H-^{1}H COSY spectrum of **1** in DMSO-*d*$_6$ at 600 MHz.

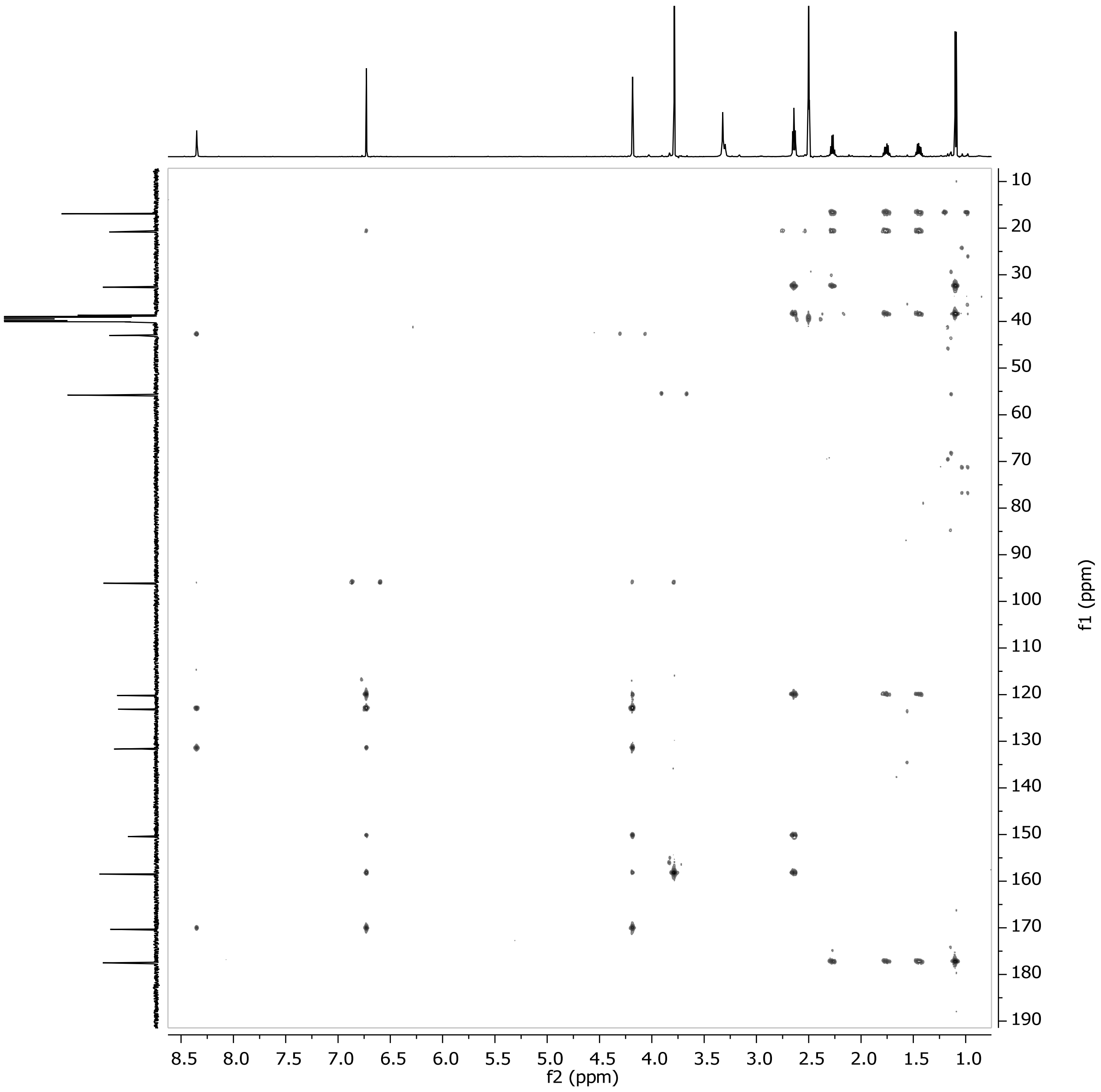

Figure S6. HMBC spectrum of **1** in DMSO-d_6 at 600 MHz.

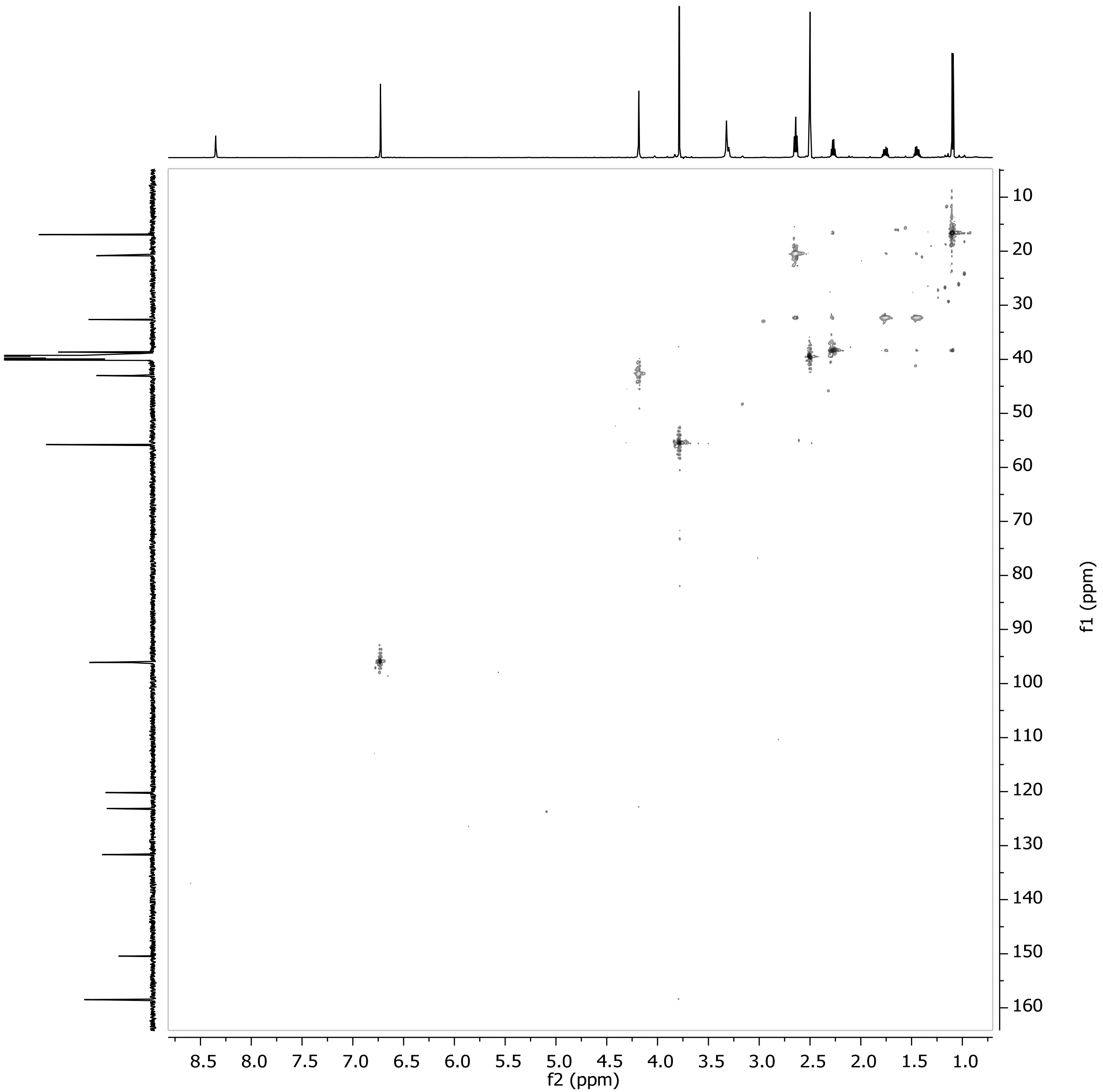

Figure S7. HSQC spectrum of **1** in DMSO-d_6 at 600 MHz.
